# Road Traffic Emissions Lead to Much Enhanced New Particle Formation through Increased Growth Rates

**DOI:** 10.1021/acs.est.3c10526

**Published:** 2024-06-08

**Authors:** James Brean, Alex Rowell, David C.S. Beddows, Kay Weinhold, Peter Mettke, Maik Merkel, Thomas Tuch, Matti Rissanen, Miikka Dal Maso, Avinash Kumar, Shawon Barua, Siddharth Iyer, Alexandra Karppinen, Alfred Wiedensohler, Zongbo Shi, Roy M. Harrison

**Affiliations:** † Division of Environmental Health and Risk Management, School of Geography, Earth and Environmental Sciences, University of Birmingham, Birmingham B15 2TT, United Kingdom; ‡ Leibniz Institute for Tropospheric Research, Leipzig 04318, Germany; § Aerosol Physics laboratory, Tampere University, Tampere 33720, Finland; ∥ Department of Environmental Sciences, Faculty of Meteorology, Environment and Arid Land Agriculture, King Abdulaziz University, Jeddah 21589, Saudi Arabia

**Keywords:** aerosol, NPF, traffic, pollution, nucleation

## Abstract

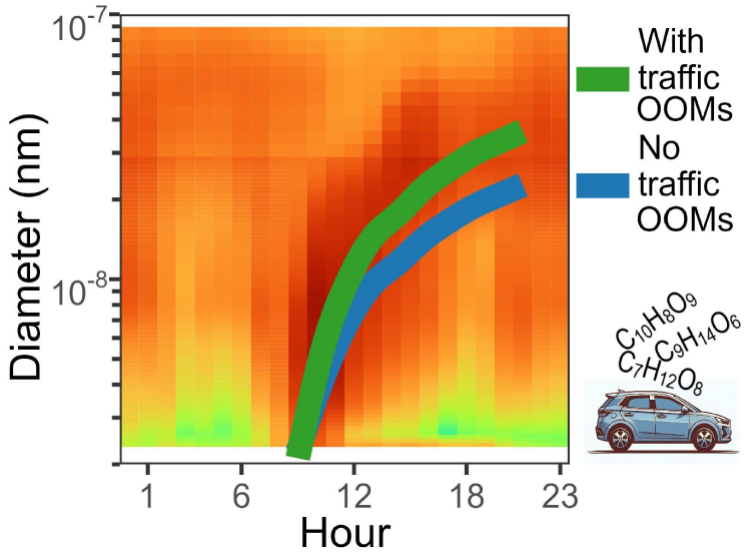

New particle formation (NPF) is a major source of atmospheric aerosol particles, including cloud condensation nuclei (CCN), by number globally. Previous research has highlighted that NPF is less frequent but more intense at roadsides compared to urban background. Here, we closely examine NPF at both background and roadside sites in urban Central Europe. We show that the concentration of oxygenated organic molecules (OOMs) is greater at the roadside, and the condensation of OOMs along with sulfuric acid onto new particles is sufficient to explain the growth at both sites. We identify a hitherto unreported traffic-related OOM source contributing 29% and 16% to total OOMs at the roadside and background, respectively. Critically, this hitherto undiscovered OOM source is an essential component of urban NPF. Without their contribution to growth rates and the subsequent enhancements to particle survival, the number of >50 nm particles produced by NPF would be reduced by a factor of 21 at the roadside site. Reductions to hydrocarbon emissions from road traffic may thereby reduce particle numbers and CCN counts.

## Abstract 2

Traffic is a source of oxygenated organic molecules, which enhance the growth rates and survival probability of new particles.

## 1 Introduction

New particle formation (NPF), the process by which gases transform into new particles, occurs ubiquitously in the boundary layer, contributes >50% to global cloud condensation nuclei (CCN) budgets,^1^ and is a substantial source of urban particulate matter.^2,3^ The ultimate climate forcing effects of these particles, as well as the health burdens from elevated PM_2.5_ mass loadings, are currently unquantified. In urban environments, newly formed particles have been shown to grow by the condensation of sulfuric acid (H_2_SO_4_) and oxygenated organic molecules (OOMs).^4−7^ These particles are highly hygroscopic,^8^ providing a potential new chemical interface for multiphase chemistry as they grow.^9^

The NPF frequency and intensity are modulated largely by the balance between the production rate of precursor vapors such as sulfuric acid,^10^ amines,^11,12^ and OOMs,^4−6,13^ and the loss of these vapors, as well as freshly formed nuclei, to pre-existing particle surfaces.^14^ Increases in temperature will increase evaporation rates of new clusters, consequently reducing formation rates;^15,16^ conversely, increases in ion pair production rates will decrease evaporation rates, increasing formation rates.^16,17^ While concentrations of vapors and particles will change substantially across an urban area, the temperature and ion pair production rates will not.

Across Europe, less frequent but more intense particle formation is observed at roadsides than urban backgrounds.^18^ Road traffic emissions are a dominant source of dimethylamine.^19^ Traffic exhaust is rich in aromatic volatile organic compounds (VOCs), and in laboratory studies, the photo-oxidation of traffic exhaust in chambers results in rapid particle formation and growth^20^ through the generation of low-volatility organic compounds (LVOCs).^21^ However, traffic is also a source of particles with a high surface area,^22^ which will act as a sink for low volatility molecules in the gas phase. Road traffic exhaust is also a source of NO_*x*_, the presence of which increases the mean volatility of OOMs produced through autoxidation,^23^ leading to slower early stage particle growth. The roadside changes in NPF frequency and intensity are therefore likely to be controlled by the interplay between these emissions of gases and particles.

The characteristics of roadside NPF are likely different to those at urban background sites. As our current mechanistic understanding of urban NPF comes from measurements of H_2_SO_4_, amines, OOMs, and their clusters conducted at urban background sites,^4−6,11,12,15,20,24^ there is a gap in the understanding of NPF and the consequences with regards to particle number, particle mass, and CCN yields. While one study has evaluated the effect of traffic emissions on NPF,^20^ no measurements of OOMs nor H_2_SO_4_ were performed. Where roadside measurements using chemical ionization mass spectrometry (CIMS) have been performed, analysis has focused on the primary emission of sub-3 nm particles^10,25^ rather than their secondary production. Furthermore, measurements in polluted urban areas are still relatively scarce compared to more remote environments,^15,17^ with little data focusing on Europe.^6^ Therefore, the available data to understand the mechanisms by which traffic influences NPF are sparse.

Understanding the future interaction between primary anthropogenic emissions and NPF is of both scientific and social interests. It has implications for the earth’s energy balance and for visibility and human health in urban areas, especially considering expected changes to tailpipe control technologies and vehicle fleets. To understand this, we must first understand the present-day influence of traffic on NPF. In this work, we deploy a suite of particle counting and nitrate CIMS instruments at an urban background and roadside site synchronously. We show that particle growth rates are enhanced at roadsides and show that this is through the formation of OOMs. We further investigate the balance between enhanced growth rates and enhanced coagulation sinks at roadsides, showing traffic emissions to be an essential ingredient of urban NPF.

## 2 Materials and Methods

All measurements were taken during a summertime field campaign in Leipzig, Germany, from 2022/08/01 through 2022/08/23. Leipzig is Germany’s eighth most populous city with ∼600 000 inhabitants in an area of 300 km^2^ and is representative of an average European urban area. All times shown throughout the paper are in local time. Extra methods are found in the Supporting Information.

### 2.1 Site Description

Measurements were taken at an urban background site and a highly trafficked roadside site. Maps and photographs of the sites are given in Figure S1. The urban background data were collected at an atmospheric research station operated by the Leibniz Institute for Tropospheric Research within the Leipzig Science Park (N 51°21′09″, E 12°26′04” 127 m above the mean sea level), hereon referred to as simply “background”. Measurements were taken out of a south-facing window on the fourth floor of a research building at 14 m above the ground level and at distances >100 m from highly trafficked roads boarding the site.^26^ The park perimeter includes transport infrastructure (road, rail, and tramways), commercial property (restaurants, hotels, a petrol station, etc.), residential property, on-street parking, and greenspace.

Roadside aerosol data were obtained from a permanent observation site located on Eisenbahnstraße, an important connecting road in the east of the city (N 51°20′44″, E 12°24′23″, 120 m above mean sea level), hereon referred to as simply “roadside”. Measurements were taken from an apartment window 6 m above ground level on the northern side of the street. The street is ∼20 m in width and is flanked by multistory period buildings, yielding an aspect ratio of 0.90, and it experiences 12,000 vehicles per working day.^26^ The stations at immediate surroundings also include two lanes of traffic (one in each direction of travel), an integrated tramline, on-street parking, two bicycle lanes (one in each direction of travel), two footpaths, and scant vegetation.

### 2.2 Size Distribution

At the background site a dual mobility particle size spectrometer (D-MPSS) collected the particle number size distribution (PNSD) from 3 to 800 nm. This system is comprised of a drier and an in-house constructed particle sizer with two differential mobility analyzer columns leading to two condensation particle counters (CPC 3025 and CPC 3010). The PNSD from 2.5 to 42 nm was also collected using a neutral cluster and air ion spectrometer (NAIS, Airel, Estonia), which also measures the PNSD of naturally charged ions from 0.8 to 42 nm. No drying was used here. The D-MPSS data have been collected at this site since 2010, while all other data were collected just for the period of the field campaign.

At the roadside site, the PNSD from 10 to 800 nm was collected using a CEN/TS 17434:2020-compliant mobility particle size spectrometer (MPSS). This comprised a drier, an in-house built particle sizer system, and a CPC 3010. The PNSD from 4.5 to 62 nm was collected using a Nano-MPSS (NanoSMPS, TSI, USA), with no drier attached to the inlet. The PNSD below this point was collected using a 3756 CPC with a 50% detection efficiency diameter (*D*_50_) of 2.5 nm (TSI, USA), and a particle size magnifier (Airmodus, Oy) attached to a 3775 CPC (TSI, USA). The particle size magnifier was run in continuous flow mode, such that the whole system has a *D*_50_ of 1.5 nm. The difference in concentration measurements between these instruments was used to measure the 2.5–4.5 and 1.5–2.5 nm fractions. The MPSS data from 10 to 800 nm have been collected at this site since 2011, while all other data were collected just for the period of the field campaign.

Data inversion and diffusive loss corrections for the D-MPSS at the background and the MPSS were done manually. Due to software constraints, the inversion and internal instrument diffusive losses for the TSI Nano-MPSS were done within the AIM10 software separately, while inlet loss corrections were done manually. For both instruments, the total counts for the short column were corrected to those for the long column by the ratio of counts at 40 nm to harmonize the size distributions.

### 2.3 Chemical Ionization Mass Spectrometry

The University of Birmingham (UoB) and University of Tampere (TAU) CIMS instruments were operated with Eisele-type inlets using nitrate charger ions to measure strong acids and oxygenated organic molecules.^27^ Both instruments were calibrated side-by-side before the campaign using the updated methodology of Mettke et al.^28^ A detailed description of the instruments and methodologies are found in Supporting Information Section 1.1.

### 2.4 Particle Formation and Growth Rates

The formation rate of new particles at size *d*_*p*_ (*J*_dp_) is calculated^29^ from the PNSD (Supporting Information Section 1.2). We use the formation rate of particles at 5 nm here, using the size bins from 5 to 10 nm. This is denoted as *J*_5_. The GRs used to calculate *J* are those calculated using eq 1.

### 2.5 Simulated Particle Growth Rates

We estimate the rate of particle growth from the condensation of acids and OOMs from the properties of gas and particles as follows:^5,30^
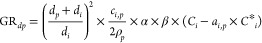
1

In this equation, *d*_*i*_ and *d*_*p*_ are the diameters of the gas molecule *i* and particle *p,* respectively. *c*_*i*,*p*_ is the center of mass velocities of the gas and particle. *ρ*_*p*_ is the density of the particle phase. α is the mass accommodation coefficient, here presumed to be 1. β is a transition regime correction. *C*_*i*_ is the concentration of the vapor molecule *i. a*_*i*,*p*_ is the particle phase activity of the molecule *i.* A breakdown of these terms is found in the Supporting information Section 1.

In this study, we estimate the saturation vapor pressure using the method of Qiao et al.^5^ This method differentiates between the products formed by autoxidation and those formed by multigenerational OH oxidation.

### 2.6 Aerosol Box Model

We simulate the observed NPF events using a box model.^31^ The purpose of this is to gain insights into the particle formation and growth dynamics within the size range of 5 nm to larger sizes. Notably, photochemistry was deactivated for this simulation, and the particle formation rates were derived from the PNSD. We followed the same procedure as discussed in 2.5 to simulate the growth rates using the condensation of H_2_SO_4_ and OOMs.

The model is initialized with the average PNSD during NPF periods. This particle population undergoes no coagulation or growth. The newly formed particles can coagulate with both themselves and the preexisting particles. The model features 100 logarithmically spaced bins ranging from 5 nm to 1000 nm. Sizes below 5 nm were not considered in this simulation to maintain consistency with the PNSD measurements. The survival probability of particles between 5 and 10 nm is calculated as the ratio of particles formed at 5 nm to that which reaches 10 nm.

### 2.7 Positive Matrix Factorization

Positive matrix factorization (PMF) is a well-established receptor model used to solve functional mixing models when the source profiles are unknown and presumed to be constant.^32^ Here, we apply PMF to the high-resolution peak fits from the CIMS data. PMF2.exe is the program used for this work. A simple error estimate is made based on the signal intensity, and they are scaled to get a good model-fit for each factor solution number in the data. Details of the PMF methodology are found in Supporting Information Section 1.4.

## 3 Results

### 3.1 Features of NPF

NPF days were defined as those with the appearance of a new mode of particles at the smallest measured sizes that grew to larger sizes. We identified 7 NPF days across our 24 day measurement set in the PNSD data at both sites (site locations in Figure S1), giving an NPF event frequency of 29% (full PNSD contour plots and particle counts in Figure S2), while long-term analyses have shown an annual frequency of 17%, with roughly half of the events occurring in the summer months.^18^ All events happened synchronously at both the background and roadside sites. NPF events began between 08:30 and 12:30, and the visual signature of the event disappeared between 09:30 and 18:30, lasting an average of 6 h. The beginning of NPF events is typically concurrent with the morning traffic rush, rather than the midday peak in solar radiation.

Averaged across the campaign period, condensation sinks are higher at the roadside than urban background (0.023 and 0.012 s^–1^, respectively), as are particle counts (3.30 × 10^4^ and 1.12 × 10^4^, respectively), and mass concentrations obtained from the PNSD measurements (18.8 and 11.2 μg m^–3^, respectively, presuming a particle phase density of 1500 kg m^–3^). Temperatures across the campaign period were particularly high for the region (mean temperature 22.8 °C, peak temperature 37.8 °C), with mean wind speeds of 1.3 m s^–1^.

With the nitrate CIMS, we measured 604 compounds at the roadside and 583 at the background. 518 compounds are found in common. In general, these are higher in concentration at the roadside (correlations between compounds with the same chemical formulas at each site in Figure S3). OOM concentrations were greater at the roadside than those at the background (9.9 × 10^7^ cm^–3^ and 5.5 × 10^7^ cm^–3^, respectively), as are H_2_SO_4_ concentrations (mean summed H_2_SO_4_ and (H_2_SO_4_)_2_ concentrations 7.7 × 10^5^ cm^–3^ and 7.3 × 10^5^, respectively, Figure S4, campaign averages). This was most marked for the H_2_SO_4_ dimer (mean (H_2_SO_4_)_2_ concentrations 2.4 × 10^4^ cm^–3^ and 1.5 × 10^4^ cm^–3^).

OOMs at both sites cover a wide range of volatilities and likely precursors. Mass defects colored by O:C ratios, double bond equivalence (DBE) per carbon, and carbon numbers are shown in Figure S5 with volatility distributions and carbon number distributions in Figure S6. DBE values vary between zero (aliphatic) and 1 (PAHs), while carbon numbers range up to >20. These larger compounds tend to have lower DBE values. The highest carbon number compounds (>C_20_) were only found at the roadside. At both sites, OOMs with a large range of H:C ratios were observed from 0.7 through 2.4. There was little difference in the mean H:C ratios between the roadside and urban background sites (1.43 and 1.48, respectively), nor in O:C ratios (0.85 and 0.89, respectively). The volatility distributions at both sites share the same shape, and approximately 50% of all OOMs contain nitrogen at each site. The biggest difference is in the carbon number distribution, where there are markedly higher concentrations of C_6_ and C_7_ OOMs at the roadside.

*J*_5_ was greater at the roadside than the background, although at the roadside, this will be the function of both photochemical and traffic nucleation (Figure S7). *J*_5_ at both sites will be the sum of fresh 3 nm particles from traffic and 3 nm particles arising from NPF. The contribution from traffic is greater at the roadside and visible in the mean diurnal cycle in *J*_5_, where an afternoon peak distinct from NPF is observed. *J*_5_ exhibits a strong positive relationship with H_2_SO_4_ and (H_2_SO_4_)_2_ concentrations, indicating that most particles form through sulfuric acid nucleation, rather than the nucleation of organic vapors leaving the tailpipe of vehicles (Figure S7).

### 3.2 The Enhancement of GRs at the Roadside

**Figure 1:**
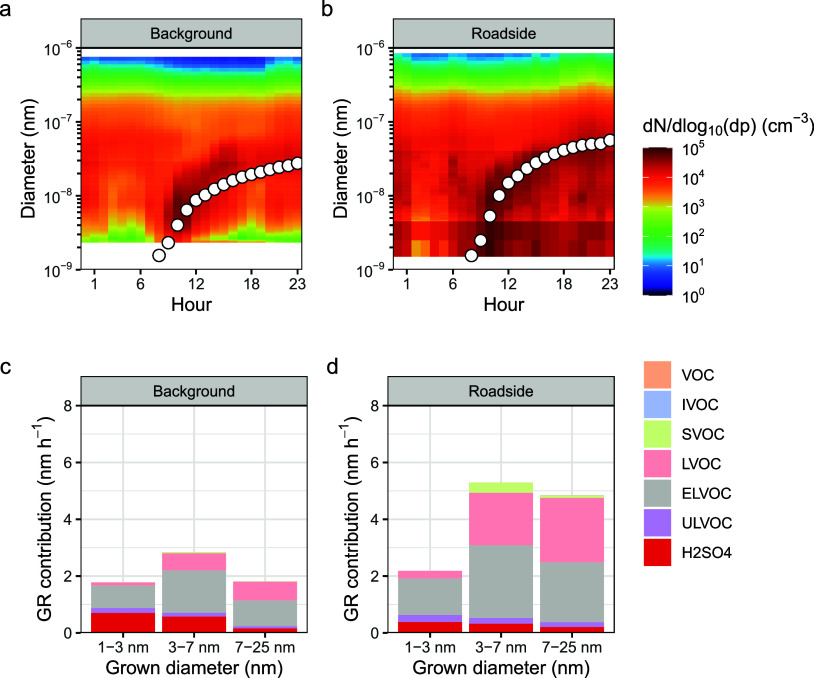
Average simulated particle growth averaged across all NPF days, showing the mean diurnal cycle in the particle number size distribution, with the simulated particle growth plotted in white over the top at the background (a) and roadside (b) sites, and the contribution of different species to particle growth averaged across all NPF days at the background (c) and roadside (d) sites. ULVOC stands for ultralow volatility organic compounds, ELVOC for extremely low volatility organic compounds, LVOC for low-volatility organic compounds, SVOC for semivolatile organic compounds, IVOC for intermediate-volatility organic compounds, and VOC for volatile organic compounds. Acids include the sulfuric acid monomer and dimer, as well as the contributions of HIO_3_ and MSA, although their contributions were low.

To understand the drivers of particle growth, we simulated the growth of a freshly formed particle starting at 1.5 nm through the condensation of acids and OOMs as measured by the nitrate CIMS for each NPF day at each site (average growth of a new particle plotted in Figure 1a, select day in Figure S8). Growth rates are higher at the roadside site than those at the background across all size ranges (Figure 1b) and are driven by OOMs. The simulated growth rates match those from the MPSS reasonably well given measurement uncertainties (Figure S9). Following convention, OOMs are separated into volatility classes: those important for particle growth are low volatility organic compounds (LVOC, 3 × 10^–5^ < C* < 0.3 μg m^–3^), extremely low volatility organic compounds (ELVOC, 3 × 10^–9^ < C* < 3 × 10^–5^ μg m^–3^), and ultralow volatility organic compounds (ULVOC, C* < 3 × 10^–9^ μg m^–3^). Particle growth averaged across both sites and across size range 1.5–25 nm was driven by OOMs (95%), primarily the ULVOCs (19% contribution), ELVOC (39% contribution), and LVOC (34% contribution), with a smaller contribution from H_2_SO_4_ (5%) and negligible contributions from HIO_3_ and methanesulfonic acid (MSA, size-segregated growth rate contributors plotted in Figure 1). The latter three of these species were all presumed to condense irreversibly, while the OOMs condensed reversibly based on estimated saturation vapor pressures. The contribution from these acids and ULVOC is greatest at the smallest diameter, as they are sufficiently involatile to overcome the barrier posed by the Kelvin effect. For particles of larger diameters, the contribution from the more abundant ELVOC and LVOC increases as the magnitude of the Kelvin effect is lesser.^30^

### 3.3 The Roadside Sources of OOMS

We used PMF to disentangle the different sources of OOMs in our data sets. Here, the implementation of PMF differs from that in the literature where an uncertainty matrix is constructed based on a priori knowledge of the errors in instrumentation and the correct number of factors is that which reaches the optimum Q·Qtheory^–1^ of unity.^33,34^ We similarly generate an uncertainty matrix, and we scale it to reach an optimum Q·Qtheory^–1^ for each factor number and then choose the appropriate number of factors based on the cogency and spatiotemporal behavior of the factors. This circumvents the previous limitation where high factor numbers must be chosen but were not easily physically interpretable.

**Figure 2:**
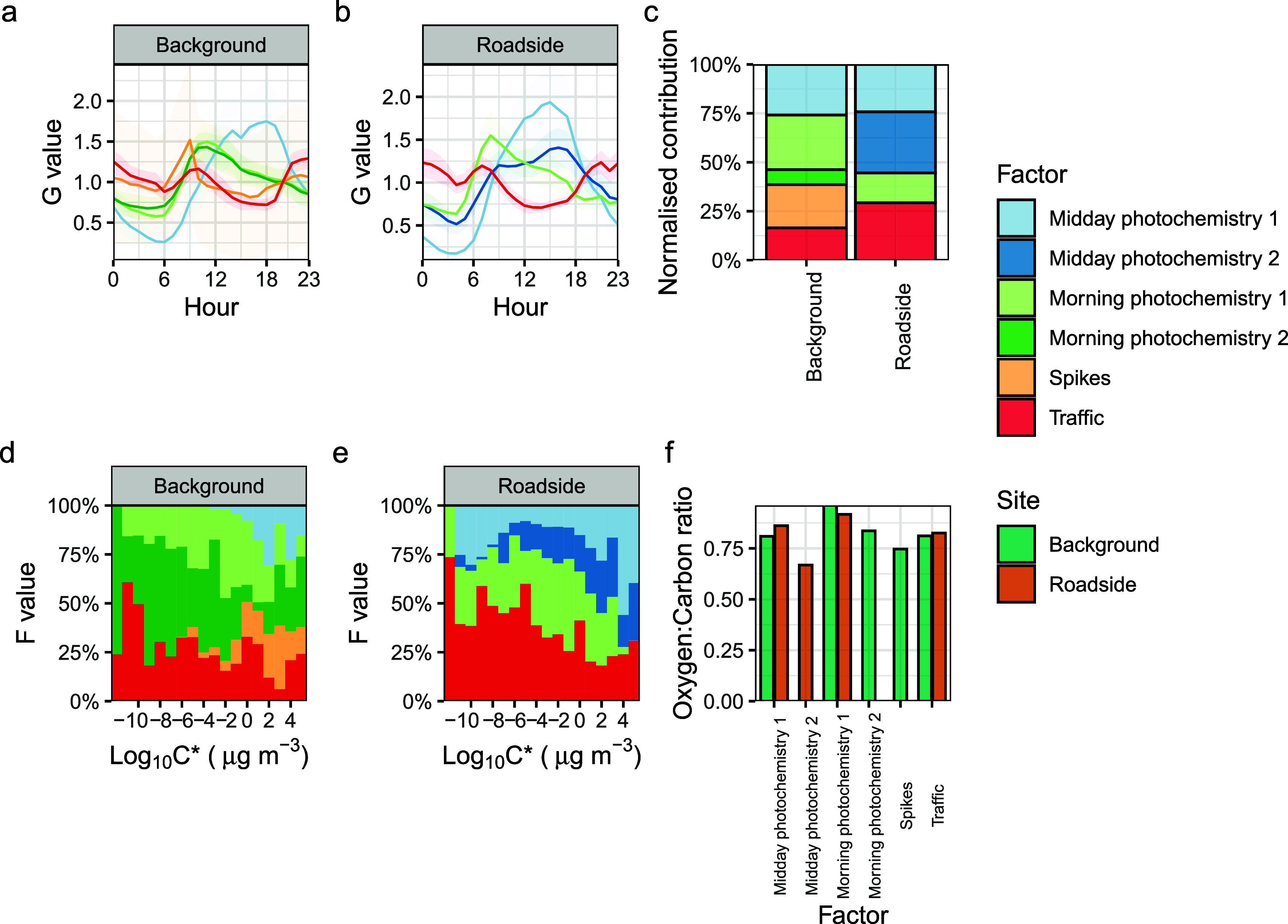
Sources of OOMs from PMF analyses. Diurnal profiles of different OOM sources at urban background (a) and roadside sites (b), contributions of each factor to the total OOM signal (c), volatility distribution of OOMs per factor for the background (d) and roadside (e) sites, and the mean O:C ratio per factor per site (f).

Through this method, we pick apart four factors associated with daytime photochemistry: one associated with occasional OOM spikes in the time series, and one factor associated with traffic (Figure 2a,b, mass spectra in Figure S10). The traffic factor at each site peaks in the morning, afternoon, and nighttime periods and is highly associated with BC and NO_*x*_ concentrations (Figure S11). The relative contribution of midday photochemistry 1 is the same at both sites, with peaks in the afternoon time. The urban background then has two morning photochemistry factors with a morning-time peak. The roadside has a midday photochemistry 2 factor with a small morning and large afternoon peak and one morning photochemistry 1 factor with a morning-time peak. The contributions of these factors differ at each site. The urban background site has a factor with large time series which here we just call spikes, attributable to a local point source. The contribution of traffic factors at the roadside site is greater than at the urban background site by around a factor of 2 (29 and 16% contributions to total signal respectively, Figure 2c).

**Figure 3:**
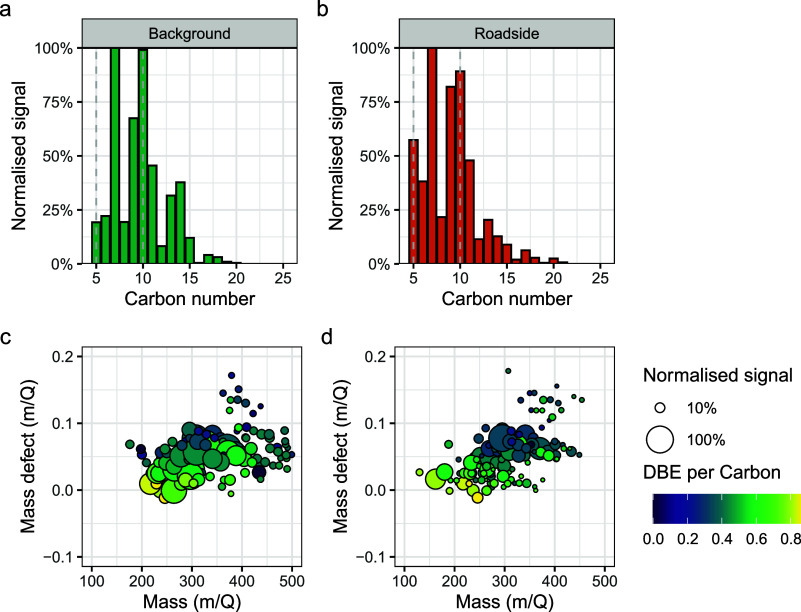
Traffic factor, showing the carbon number distribution at (a) the roadside and (b) the background sites, and the mass defect plot for the top 25% of signals in the factor at (c) background and (d) roadside sites. Mass defect is defined as the mass of an ion minus its nearest integer mass. Signals have been normalized for ease of visual comparison.

In relation to the photochemistry factors, the molecules in the traffic factors were larger, with higher average numbers of carbon, hydrogen, nitrogen, and oxygen (individual concentration weighted CHON numbers plotted in Figure S12), with the lowest carbon and nitrogen contents at both sites belonging to midday photochemistry 1. The volatility distributions for the traffic factors show peaks at 10^–8^ and 10^–5^ μg m^–3^ in the ELVOC–LVOC region. At both sites, the traffic factor is a major contributor to the least volatile OOMs, especially at the roadside (Figure 2d,e). Where factors are found in-common at each site, the O:C ratios are similar (Figure 2f). At both sites, the greatest contributors to signal were molecules with 7, 10, and 9 carbon atoms, in that order (Figure 3a,b). The mass defects show multiple homologous series of molecules, notably C_7_H_8–15_N_0–1_O_4–11_ and C_10_H_13–17_N_0–1_O_4–12_, with the vast majority of these containing nitrogen. The traffic factors are therefore similar in temporal variability and chemical composition at both sites, with the main difference being the greater contribution at the roadside site.

### 3.4 Traffic-enhanced NPF

**Figure 4:**
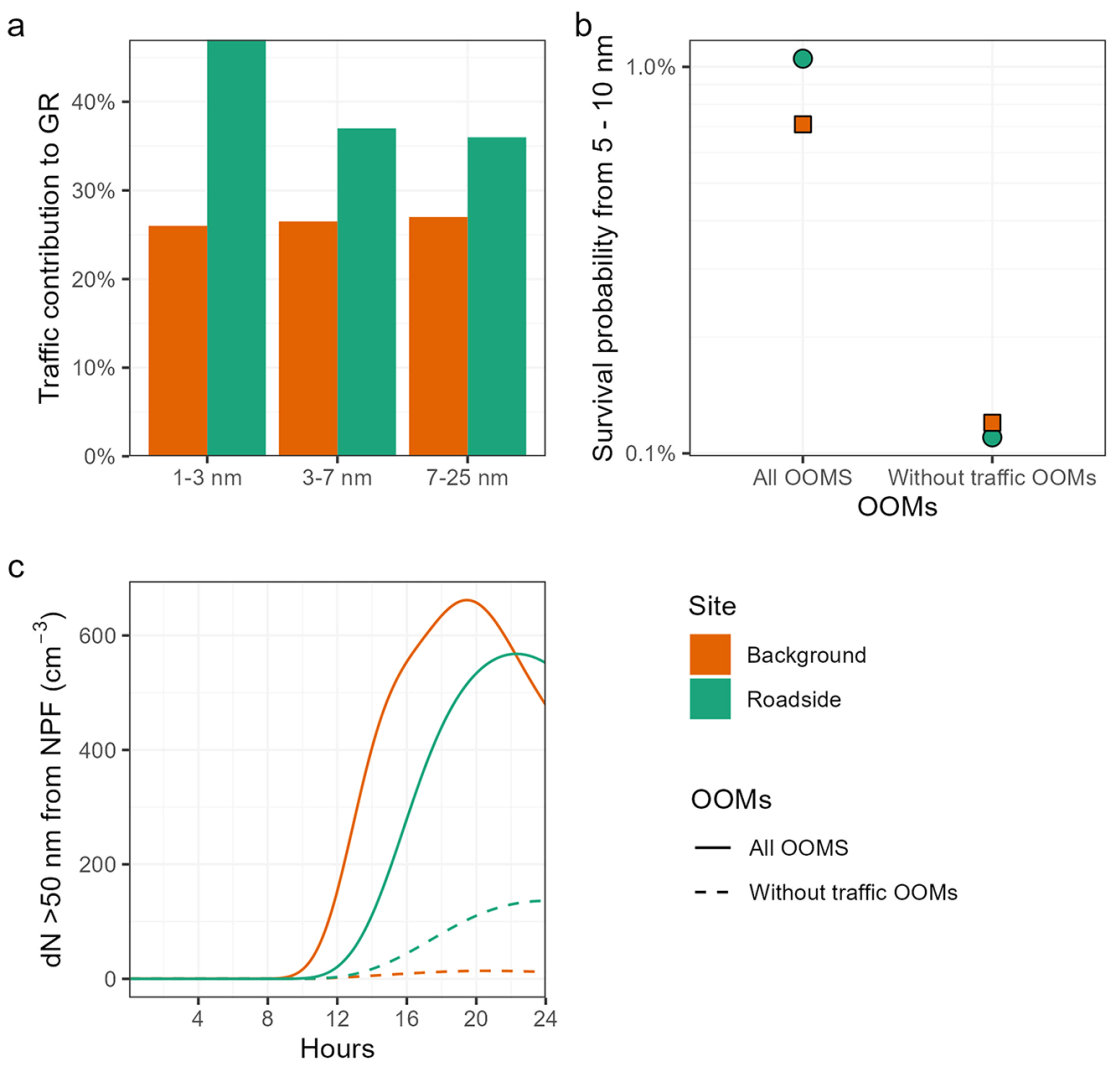
Effects of traffic OOMs on NPF. The contribution of traffic OOMs to overall GRs (a), the effect of this GR on the survival probability of new particles (b), and the effect on the resultant concentration of particles >50 nm (c).

We simulated the observed NPF events using a custom aerosol sectional box model that solves the formation, growth, and coagulation of new particles (Figure 4). Here, all simulations begin at 5 nm to keep consistency between our two data sets and to avoid dealing with uncertainties about sub-5 nm growth and coagulation rates. We conducted two sets of simulations: the first set simulated NPF at both sites with all OOMs included. For the second set, we eliminated the traffic factor altogether, thereby removing the effects of traffic-generated OOMs from particle growth. We show that despite making up <30% of total OOMs at the roadside, the contribution to growth is above 40% at the smallest sizes (Figure 4a). This can be explained by the shape of the volatility distribution, which is dominated by the species of lowest volatilities (Figure 2e). The contribution of traffic-derived OOMs is less pronounced at the background site, contributing ∼25% to GRs (Figure 4a).

The likelihood of survival of a particle depends on the balance between growth and coagulation. The increase in growth rates due to traffic OOMs increases the survival probability of new particles by a factor of 6 at the background and a factor of 12 at the roadside site (Figure 4b). The number of >50 nm particles derived from NPF (*N*_>50_) reduces drastically when the effect of traffic OOMs is removed (Figure 4c). At the 14th hour, roughly 6 h after the onset of NPF, *N*_>50_ is reduced from 1219 to 56 cm^–3^ at the roadside and from 304 to 41 cm^–3^ at the background site. At the roadside, this effectively inhibits NPF.

## 4 Discussion

### 4.1 NPF in Leipzig

Our measured NPF events are typical of a summertime European location^18^ occurring at both higher CS and J than clean remote environments,^17^ but lesser than those in polluted Chinese megacities.^15,17^ The visible signature of the NPF events we measure lasts an average of 6 h, after which it typically disappears and is indistinguishable from the background particle population. This disappearance can be attributed to two primary factors: dilution of the particles due to air mass changes or losses due to coagulation. The mean wind speed during the campaign was 1.3 m s^–1^. If the visual signature disappears due to air mass dilution, we can infer that the NPF event occurs over a range of, on average, 30 km. Long-term observations have shown that around half of summertime NPF events have been shown to occur synchronously at this site and a site 50 km away.^18^ The NPF events we observe therefore tend to occur on a relatively small scale around Leipzig.

Measured GRs in our data set are 3 nm h^–1^ greater at the roadside than those at the background site. This is in line with long-term data at these sites, with similar enhancements seen across Europe.^18^ We have shown that particle growth in our data set is driven largely by the condensation of OOMs, with a small fraction of growth through condensation of H_2_SO_4_ and a negligible contribution from the typically marine-sourced MSA and iodine oxoacids (Figure 1). The composition of new particles inferred from condensation is consistent with that directly measured using thermal desorption mass spectrometry techniques in other studies.^4,35^ This, along with the consistency between the measured and modeled growth rates in both these studies and our own confirms that the measurement and theoretical work here are sufficient to capture the particle growth mechanisms in the urban environment. Particle growth is therefore driven largely by OOMs that derive from both biogenic and anthropogenic sources, of which approximately half contain nitrogen, indicating a role of NO_*x*_ in oxidation chemistry.^23^ This mechanism of particle growth is therefore accurate and likely representative of urban Central Europe at large where both vehicle fleets and biogenic emissions show major similarities between cities.

### 4.2 Traffic OOMs Boost Urban NPF

Our PMF results indicate a source of OOMs, which peaks in traffic hours, is highly correlated with the primary traffic pollutants NO_*x*_ and BC and contributes more to OOM signals at the roadside site (Figures 2 and S11). Autoxidation is well-known to take place efficiently in low-temperature combustion systems^36−39^ with OOM yields correlating positively with temperature.^40^ We therefore identify a previously unreported source of OOMs, which we will henceforth refer to as traffic OOMs. The source may be primary or the result of a rapid atmospheric oxidation.

Our observed traffic OOMs may either be primary, arising from oxidation before vapors leave the tailpipe, or secondary, deriving from the rapid oxidation of VOCs emitted from vehicles. Autoxidation happens on a time scale of milliseconds and becomes more rapid with increasing temperature,^41^ making engines prime autoxidation locations. Recent laboratory experiments show that alkylperoxy radicals formed in low-temperature ignition also form oxygenated products with a high O:C ratio in the particle phase. These alkylperoxy radicals will also be responsible for autoxidation resulting in OOMs measurable by the nitrate CIMS, and these would be primarily emitted from the exhaust.^42^ However, engine exhaust is rich in aromatic VOCs.^20^ It is known that the oxidation of single ring aromatics and smaller PAHs produces OOMs under ambient conditions through autoxidation^43,44^ as well as multigenerational OH• oxidation.^21,45^ Engine exhaust is also rich in alkanes,^46^ and straight-chain compounds have been shown to produce OOMs efficiently, even under high NO_*x*_ conditions,^39^ with similar products measured across China.^47^ Furthermore, nonexhaust VOCs from screenwash have recently been shown to be a major VOC source from traffic, although they tend to be small alcohols.^48^ Our traffic factor is dominated by alkylbenzenes and possibly either monoterpene or C_10_ alkylbenzene oxidation products (Figures 3 and 4). As the diurnal cycle of this source directly tracks traffic emissions (Figures 2a,b and S11), it is likely to be primarily emitted. The high temperatures in traffic exhaust and resultant rapid autoxidation may explain the low volatility of the traffic OOMs.^41^

Traffic OOMs have, on average, a sufficiently low volatility to drive particle growth and in fact make up a large portion of ELVOC and ULVOC concentrations (Figure 2). These contribute disproportionally to growth rates due to the shape of this volatility distribution. Coagulation rates are highest for small particles, with the growth between the 1.5 and 10 nm fraction being dubbed the “valley of death”.^49^ For particles to pass this valley they need to grow sufficiently fast. Our box modeling results (Figure 4) demonstrate that these traffic OOMs increase the survival probability of particles growing from 5 to 10 nm by an order of magnitude. This then results in a factor of 20 decrease to new particle number concentrations >50 nm from NPF at the roadside and in a factor of 7 decrease at the urban background when this source of OOMs is removed. These traffic OOMs are therefore an essential component of urban NPF.

### 4.3 Uncertainties and Assumptions

CIMS instruments have differing sensitivities depending on reagent ion distributions and instrument tuning. We colocated our instruments for calibrations and voltage tunings, producing instruments with similar distributions of the charger nitrate ion monomer (NO_3_^–^), dimer (HNO_3_NO_3_^–^), and trimer ((HNO_3_)_2_NO_3_^–^). In our measurement results the ratio of CIMS concentrations between the two sites has a consistent ratio at different masses (Figure S3), and therefore, there is no bias toward more oxygenated species between either instrument. Our instruments both therefore are operating similarly.

Previous studies focusing on particle growth^4,35^ deal with a particle growth of <25 nm. At diameters >25 nm, a role of more complex solute phase chemistry with salt formation and oligomerization processes in particle growth becomes likely, which we do not capture in this work.^9^ Ignoring coagulational growth, the particle growth rate is a competition between condensation and evaporation. The abundance of gases largely governs the former, while the volatilities of the involved species are more important governing the latter.^50^ The assumptions made in our estimation of saturation vapor pressure, that is, the assumed functional groups, are a great source of uncertainty in these calculations, with orders of magnitude difference between different methods of measuring and estimating saturation vapor pressure.^51^ We also presume that particle phase reactions cause no change in the partitioning, although these reactions are relatively fast and likely result in a net decrease in evaporation rates of condensed phase OOMs.^52^ The nitrate CIMS lacks sensitivity to certain key OOMs, such as those with only one hydrogen bond donor group,^27^ and our OOM concentrations are therefore a lower limit. We present our nitrate CIMS measurements with approximate measurement uncertainty, which is estimated to be +80%/–45% for OOMs and +50%/–33% for sulfuric acid, given uncertainties related to calibration. We also neglect the coagulational growth contributed from primary particle emissions, and we calculate the coagulational growth to be around ∼10% of the total growth rate at both the roadside and background site. We also acknowledge that J_5_ here is the sum of both J from NPF and also J from primary and delayed primary traffic particles; however, MPSS instruments cannot distinguish new particles from either source. As the primary and delayed primary source is strongest at the roadside, we acknowledge a bias in total particle numbers in Figure 4; however, the changes to survival probability of new particles are mostly independent of J. Finally, we neglect any role of ammonium nitrate in the growth modeling, as chamber work shows that this process is highly temperature dependent and likely to be significant only at temperatures below those of this campaign.^49,53^ Despite this, our modeled condensation rates match the evolution of the PNSD. We therefore capture the processes most relevant to urban particle growth.

### 4.4 Implications and Future Perspectives

We show that traffic OOMs are essential for the growth and therefore survival of new particles to larger sizes. Notably, we emphasize that without traffic OOMs, the yields of 50 nm particles would decrease substantially. Particles must be sufficiently hygroscopic, as well as sufficiently large to be CCN active. Both sulfate and condensed-phase OOMs are highly hygroscopic.^8^ Previous measurements at the nearby station, Melpitz, have shown that during NPF events, the critical diameter for CCN activation lies between 50 and 80 nm at supersaturations between 0.4 and 0.6%.^54^ Our N_50_ can therefore represent an upper limit for CCN production, and we suggest that enhanced growth of new particles through traffic OOMs assists the production of CCN by contributing to the diameter and hygroscopicity of new particles, explaining in part the anthropogenic enhancement to CCN production.^55^

This mechanism by which traffic emissions locally enhance particle number concentrations may also be detrimental to human health,^56^ as NPF can be responsible for billions of particles depositing in the lung every NPF day.^57^ However, the main loss process for OOMs is condensation,^27^ and this occurs on the order of minutes in the urban environment for the least volatile OOMs. Sharper OOM gradients are therefore expected around roadsides than for other pollutants,^58,59^ and this explains the factor of 2 decrease in traffic OOM signals between our roadside and urban background sites. This particle number enhancement is likely therefore localized around densely populated urban areas, where any health effects will be concentrated.

Modeling studies have shown that the future magnitude of NPF is highly dependent on precursor emissions even when accounting for substantial changes to CS.^31^ Traffic emissions consist largely of gases that accelerate NPF (ammonia, amines, and now OOMs). As exhaust abatement on combustion vehicles advances and the vehicle fleet further electrifies, the concentrations of these gases are expected to fall; therefore, reductions to the numbers of CCN arising from urban emissions are expected. This will lead to changes in cloud albedo and lifetime,^60^ potentially affecting surface warming.

## Supporting Information Available

Extended details of the methodology, location of measurement sites (Figure S1); PNSD at both sites for the whole measurement period (Figure S2); concentrations of all species (Figure S3); relative mean concentrations of OOMs and H_2_SO_4_ (Figure S4); mass defect plots of all identified molecules (Figure S5); volatility and carbon number distribution of OOMs (Figure S6); sulfuric acid and formation rates (Figure S7); example simulated particle growth (Figure S8); GRs from fitting modes to the average of MPSS data and from the calculated GR from the CIMS data (Figure S9); average mass spectrum (Figure S10); association of BC and NO_*x*_ with each PMF factor at each site (Figure S11); average signal-weighted carbon, hydrogen, nitrogen, and oxygen numbers per PMF factor per site (Figure S12); calibration run results for both instruments (Figure S13) (PDF)
