# Supplementary material for: Road Traffic Emissions Lead to Much Enhanced New Particle Formation through Increased Growth Rates
Source: Environ Sci Technol. 2024 Jun 8;58(24):10664–74. doi: 10.1021/acs.est.3c10526 (PMC11191591; doi:10.1021/acs.est.3c10526)
Supplement: Supplementary file 1 — es3c10526_si_001.pdf [file es3c10526_si_001.pdf]

Supplementary information for:

Road traffic emissions lead to much enhanced new particle formation through increased growth rates

James Brean[1], Alex Rowell[1], David C.S. Beddows[1], Kay Weinhold[2], Peter Mettke[2] Maik Merkel[2], Thomas Tuch[2], Matti Rissanen[3], Miikka Dal Maso[3], Avinash Kumar[3], Shawon Barua[3], Siddharth Iyer[3], Alexandra Karppinen[3], Alfred Wiedensohler[2], Zongbo Shi[1], Roy M. Harrison[1,4*]

Corresponding author: Roy M. Harrison

Email: r.m.harrison@bham.ac.uk

Summary: 18 pages, 12 figures.

1. Methodology

1.1 Chemical ionisation mass spectrometry

The Nitrate CIMS can is highly sensitive to strongly acidic compounds and compounds with two hydrogen bond donor groups in the gas phase. This comprehensively covers most low volatility compounds in the urban atmosphere and is a suitable instrument for measuring NPF precursors in-field. The front end consists of a chemical ionisation system where a ca. 10 L min^{-1} sample flow is drawn in through the 1 m length 3/4” OD stainless steel tubing opening, where it enters the chemical ionisation chamber. Inside the chemical ionisation chamber, a secondary flow was run parallel and concentric to this sample flow, rendering the reaction chamber effectively wall-less. A 3 cm^3 min^{-1} flow of a carrier gas (N_2) is passed over a reservoir of liquid HNO_3, entraining vapour which is subsequently ionised to NO_3^- via an X-ray source. Ions are then guided into the sample flow. The nitrate ions will then charge molecules either by clustering or proton transfer. The mixed flows travelling at 10 L min^{-1} enter the critical orifice at the front end of the instrument at 0.8 L min^{-1} and are guided through a series of differentially pumped chambers before reaching the ToF analyser. ToF data analysis was carried out in the Tofware 3.2.5 package in Igor Pro 9 (Tofwerk AG, Switzerland). The limited mass spectral resolution (m dm^{-1} 3500 at m Q^{-1} 201) combined with mass calibration uncertainties makes assigning compounds >500 m Q^{-1} highly uncertain, and these were left out of the analyses. Dried & filtered compressed air was used for the sheath flows.

The two instruments were calibrated adjacently with respect to sulphuric acid [1] (Figure S10). The quantification of sulphuric acid in the CIMS is as follows

$$[H_2SO_4] = C \times (1 + \ln (\frac{H_2SO_4NO_3^- + HSO_4^-}{\sum_{n=0-2}(HNO_3)_nNO_3^-}) \quad (1)$$

And the quantification of OOMs is as follows

$$[OOM] = C \times (1 + \ln (\frac{OOM\text{−}NO_3^- + OOM^-}{\sum_{n=0-2}(HNO_3)_nNO_3^-}) \quad (2)$$

Where C is a calibration constant, here, $2.05 \cdot 10^9$ cm^{-3} for the UoB instrument and $1.07 \cdot 10^9$ for the TAU instrument. Presuming that all collisions between analyte *A* and reagent ion result in charging via either clustering or deprotonation, the production of charged analytes will continue at the kinetic limit for both H_2SO_4 and OOMs, and therefore the same *C* applies in both cases. This introduces some uncertainties, as it relies upon both collision rates and charging efficiencies to be the same within the ionisation source for all species. Blanks were performed on both instruments mid-campaign and were negligible for all compounds of interest.

1.2 Particle formation and growth rates

The formation rate of new particles at size d_p (J_{dp}) is calculated as follows [2].

$$\boldsymbol{J_{d_p} = \frac{dN_{d_p}}{dt} + CoagS_{d_p} . N_{d_p} + \frac{GR}{\Delta d_p} . N_{d_p} \quad (3)}$$

where the first term on the right-hand side comprises the rate at which particles enter the size d_p, and the second term refers to losses from this size by coagulation, $CoagS_{dp}$ being the coagulation sink at size d_p [3], and N_{dp} being the number of particles at size d_p, with the third term referring to losses from this size by growth, where the growth rate of new particles can be calculated from the PNSD as follows [2]

$$GR = \frac{ddp}{dt} \quad (4)$$

In the instance of this work, the growth rates used to calculate J are calculated from the CIMS data. We use the formation rate of particles at 5 nm here, using the size bins from 5 – 10 nm. This is denoted J_5.

1.3 Simulated particle growth rates

The rate of particle growth from the condensation of acids and OOMs can be estimated as follows [4, 5]

$$GR_{dp} = (\frac{d_p+d_i}{d_i})^2 \times \frac{c_{i,p}}{2\rho_p} \times \alpha \times \beta \times (C_i - a_{i,p} \times C^*{}_i) \ (5)$$

Where d_i and d_p are the diameters of gas molecule i and particle p respectively, c_{ip} is the centre of mass velocities of gas and particle

$$V_{i,p} = \sqrt{8RT/\pi(M_i M_p/(M_i + M_p)} \ (6)$$

ρ_p is the density of the particle phase, α is the mass accommodation coefficient, here presumed to be 1, β is a transition regime correction, C_i is the concentration of vapour molecule i, $a_{i,p}$ is the particle phase activity of molecule i, calculated as follows

$$a_{i,p} = X_{i,p} K_{dp} \gamma_{i,p} \ (7)$$

Where $X_{i,p}$ is the mass fraction of species i in the particle phase, K_{dp} is the kelvin coefficient at particle size d_p, calculated as follows

$$K_{dp} = \exp\left(\frac{4\sigma_p M_i}{RT\rho_i d_p}\right) \ (8)$$

Where σ_p is the surface tension of the particle p, here taken to be 0.044 N m^{-1} [6], M_i is the mass of species i, R is the gas constant, and T is temperature. $\gamma_{i,p}$ is the mass-based activity coefficient in the organic condensed phase, here presumed to be 1. C^*_i is the saturation vapour pressure of species i. Here, we use the method of Qiao et al. [4] to separate the calculation of products formed by autoxidation and those formed by multi-generational OH oxidation. Here, we presume that each condensing H_2SO_4 molecule is clustered with one DMA molecule.

1.4 Positive matrix factorisation

Positive matrix factorisation (PMF) is a well-established receptor model used to solve functional mixing models when the source profiles are unknown and presumed to be constant [7]. PMF solutions are constrained to be non-negative, and a least squares algorithm is applied which accounts for uncertainties in the dataset, downweighing uncertain elements. PMF is therefore quantitative and identifies physically meaningful sources, the number of which may be specified by the user, and the proper number of which is determined by analysis of the results. These benefits make it suitable to source-apportion CIMS data. PMF analysis here is performed in the PMF2 program on the time series of all peaks with more than 4 carbon atoms and 3 oxygen atoms, excluding small organic acids, H_2SO_4, HIO_3, MSA, and the reagent ions, as well as other peaks such as Br^-. The mass balance as applied to mass spectral data can be described as follows:

$$X_{ij} = \sum_{k=1}^{p} g_{ik} \cdot f_{kj} + e_{ij} \ (12)$$

Where the jth compound on the ith observation is represented by x_{ij}. The first term on the right-hand side, g_{ik} represents the contribution of the kth factor (of a total of p factors) to the receptor on the ith hour. The second term, f_{kj}, represents the fraction of the total of the jth compound to the kth factor. The

third term, e_{ij}, is the residual for the measurements in the jth compound on the ith hour. Here, the **G** matrix represents the time-series of the strength of source *k*, and the **F** matrix represents the mass spectrum produced from source *k*. The PMF program runs to minimise the sum of squared residuals scaled by their measurement uncertainty (Q), meaning the results are heavily determined by the relative input uncertainties. Q is defined as:

$$Q = \sum_{i=1}^{m} \sum_{j=1}^{n} \left(\frac{e_{ij}}{s_{ij}}\right)^2 \quad (13)$$

Where s_{ij} is the input measurement uncertainty of compound *j* at time *i*, and e_{ij} is the model residual of compound *j* at time *i*. Here, PMF is run in *robust mode*, where strong outliers with $e_{ij}:s_{ij} > \alpha$ are reduced to α. In all model runs here, $\alpha = 4$. Error estimates are not readily available for Nitrate CIMS data, so here we take the error matrix to be equal to

$$s_{ij} = \sqrt{I_{ij}} \quad (14)$$

Where *I* is the signal intensity of species *j* and time *i*. The errors for the upper and lower 5% signal quantiles were then scaled by a factor of 3 to remove the possibility of the PMF solution being driven by outliers. To interpret the association of the PMF factors with primary pollutants, we also attached the time series of BC and NO_x concentrations to the data frame entering PMF, with their errors being upscaled also by a factor of 3. The remaining uncertainty matrix was then scaled to provide a ratio of the modelled Q to the expected value of Q (Q_{theory}, which is equal to the degree of freedom of the model solution) of 1±0.01 for each solution, akin to what is used in the PNSD literature [8]. Scaling of error matrices is also used to similar effect in the CIMS literature [9].

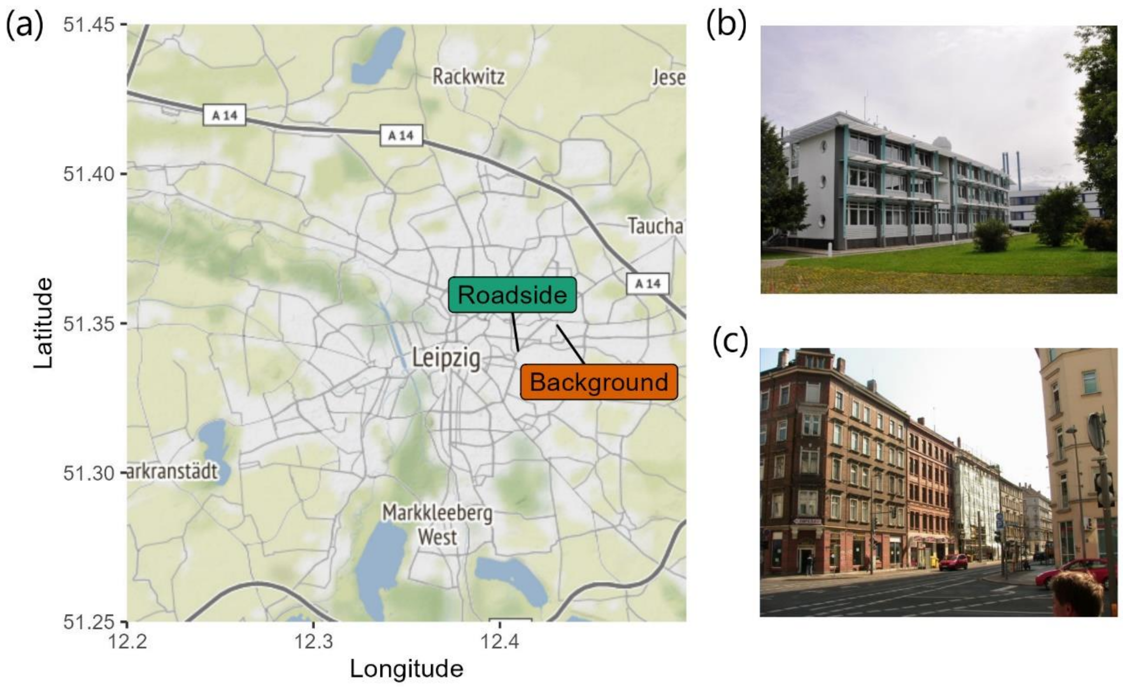

Figure S1: Location of the measurement sites, showing (a) a map of Leipzig, with the roadside and background sites labelled, (b) the background site, and (c) the roadside site. Map data from Stamen Maps, Reprinted (Adapted or Reprinted in part) with permission from ref. 10. Copyright 2016, ESSD, Copernicus.

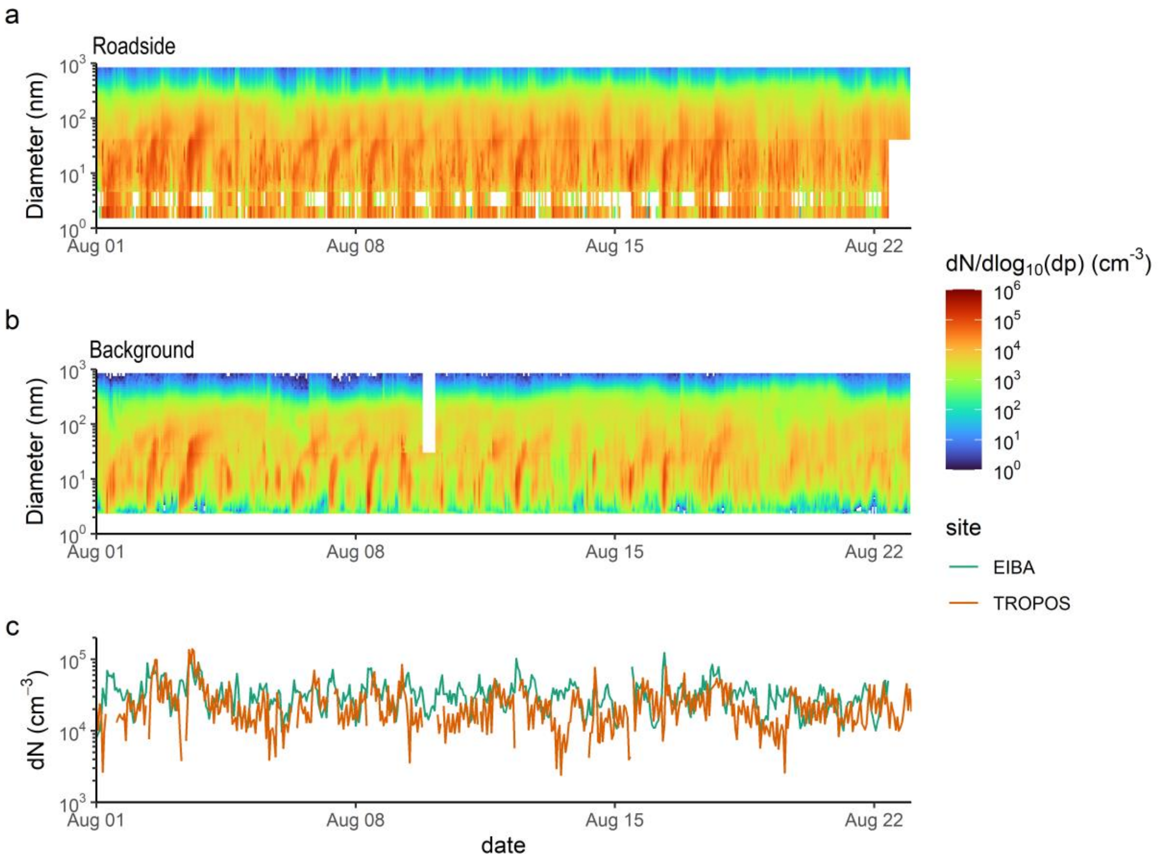

Figure S2: PNSD at both sites for the whole measurement period. The PNSD from the roadside (panel a) is generated from MPSS (10 – 800 nm) TSI NanoSMPS (4-5 – 10 nm), CPC (D_{50} 2.5 nm), and PSM (D_{50} 1.5 nm) systems. The PNSD at the background (panel b) is from a D-MPSS (10 – 800 nm) and NAIS (3 – 10 nm). The total number concentration (dN, panel c) values at the roadside are from the CPC (2.5 – 1000 nm), while the dN values at TROPOS are from integrating across the size distribution (3 – 800 nm).

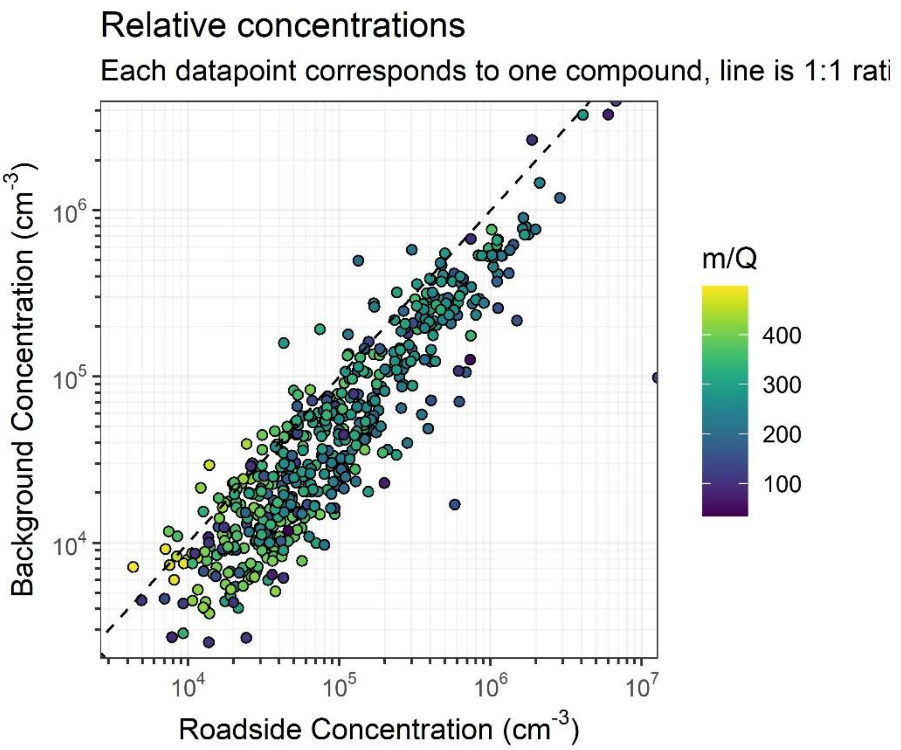

Figure S3: Concentrations of all species common to both sites at the roadside and background site. Dashed line shows the 1:1 ratio.

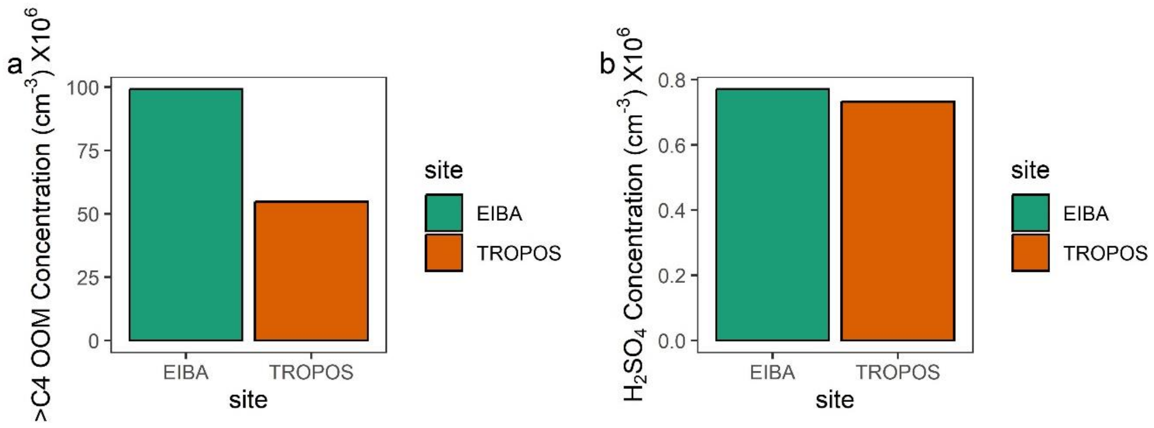

Figure S4: Relative mean concentrations of OOMs (a) and H_2SO_4 (b) at roadside and background sites.

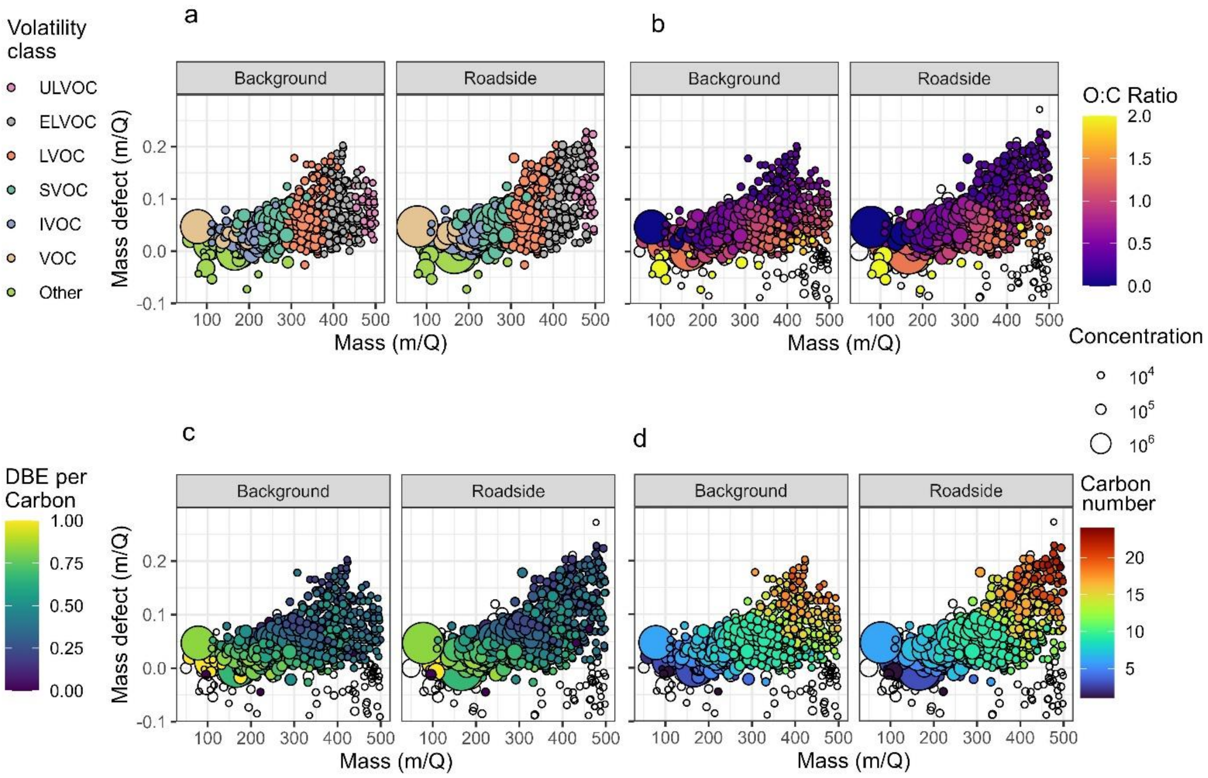

Figure S5: Mass defect plots of all identified molecules at the background and roadside sites, coloured by (a) volatility class, (b) O:C ratio, (c) double bond equivalence (DBE) per carbon, calculated as (nC + 1 – (nH – nN) / 2)/nC, where nC is the number of carbons, nH the number of hydrogens, and nN the number of nitrogens, and (d) carbon number. Mass defect is defined as the mass of an ion minus its nearest integer mass. Uncoloured points represent unidentified compounds.

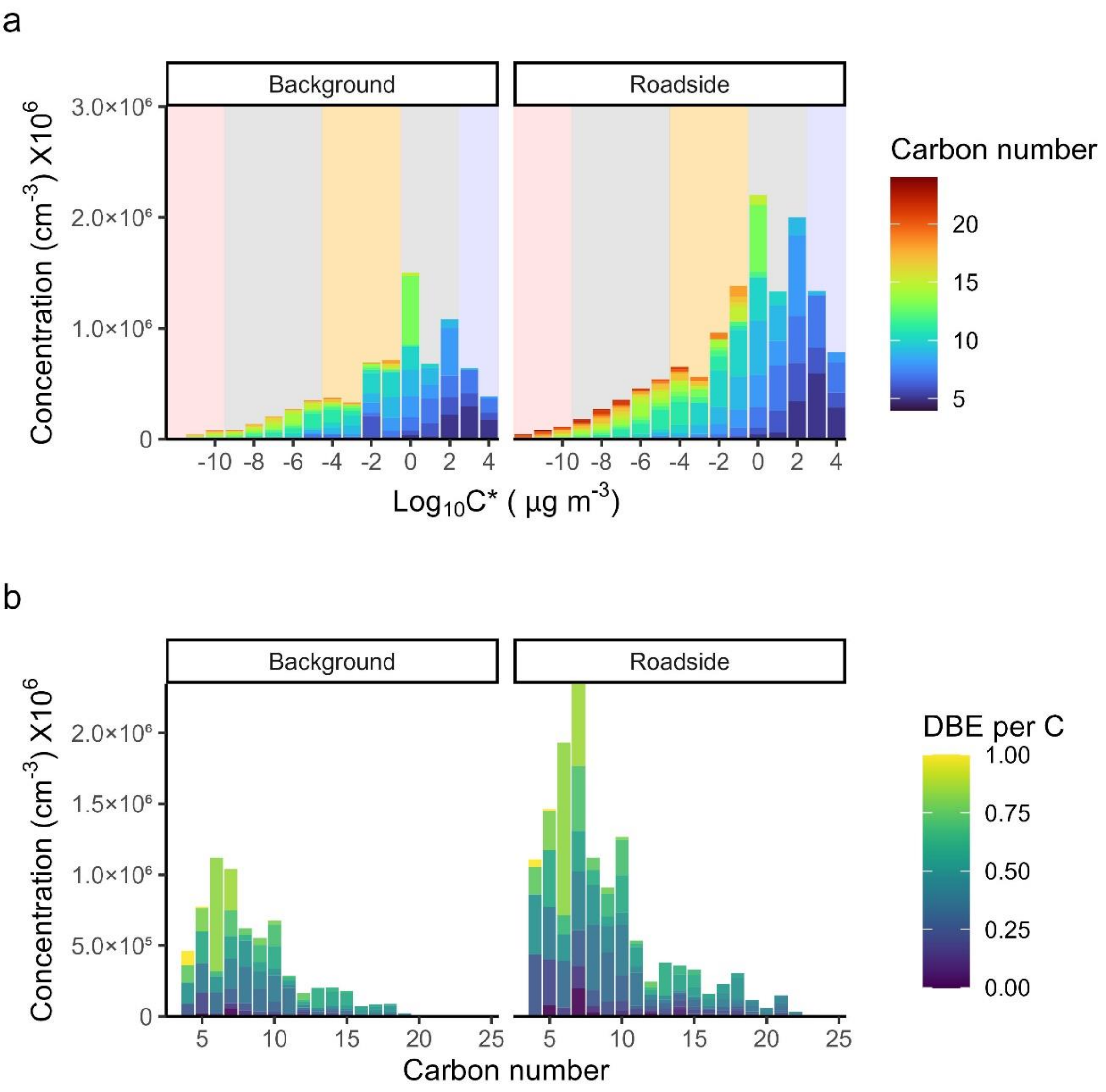

Figure S6: (a) Volatility distribution of OOMs at roadside and background sites, coloured by carbon number, and (b) the carbon number distribution of OOMs at roadside and background sites, coloured by DBE per carbon.

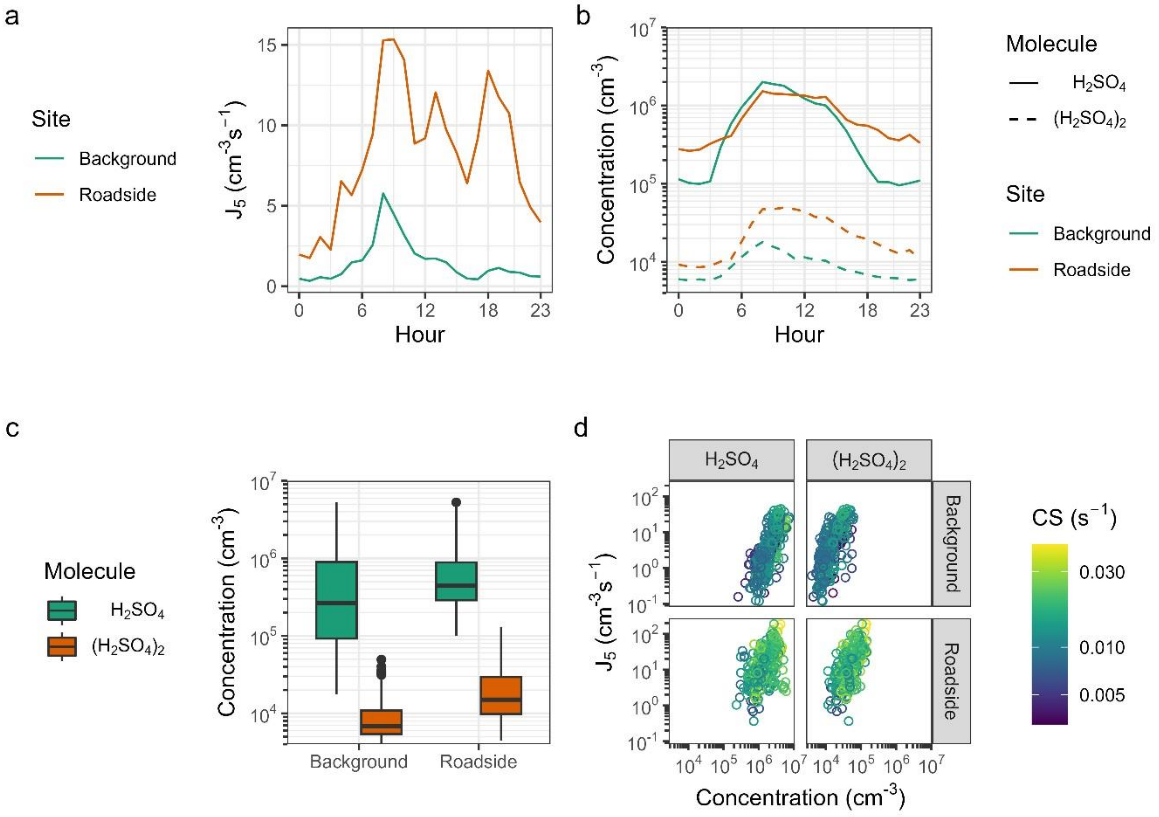

Figure S7: Sulphuric acid and formation rates at the roadside and background sites, showing (a) the diurnal cycles of H_2SO_4 and H_2SO_4 dimer at each site, (b) the diurnal variation in J_3 at both sites, (c) the concentrations of H_2SO_4 and H_2SO_4 dimer as a boxplot at each site, and (d) scatterplots of J_3 vs H_2SO_4 and H_2SO_4 dimer at each site. Box plots show median (center line), upper and lower quartiles (box limits), 1.5 times the interquartile range (whiskers), and any outliers as points. H_2SO_4 was detected as HSO_4^- and $H_2SO_4NO_3^-$, $(H_2SO_4)_2$ was detected as $HSO_4H_2SO_4^-$.

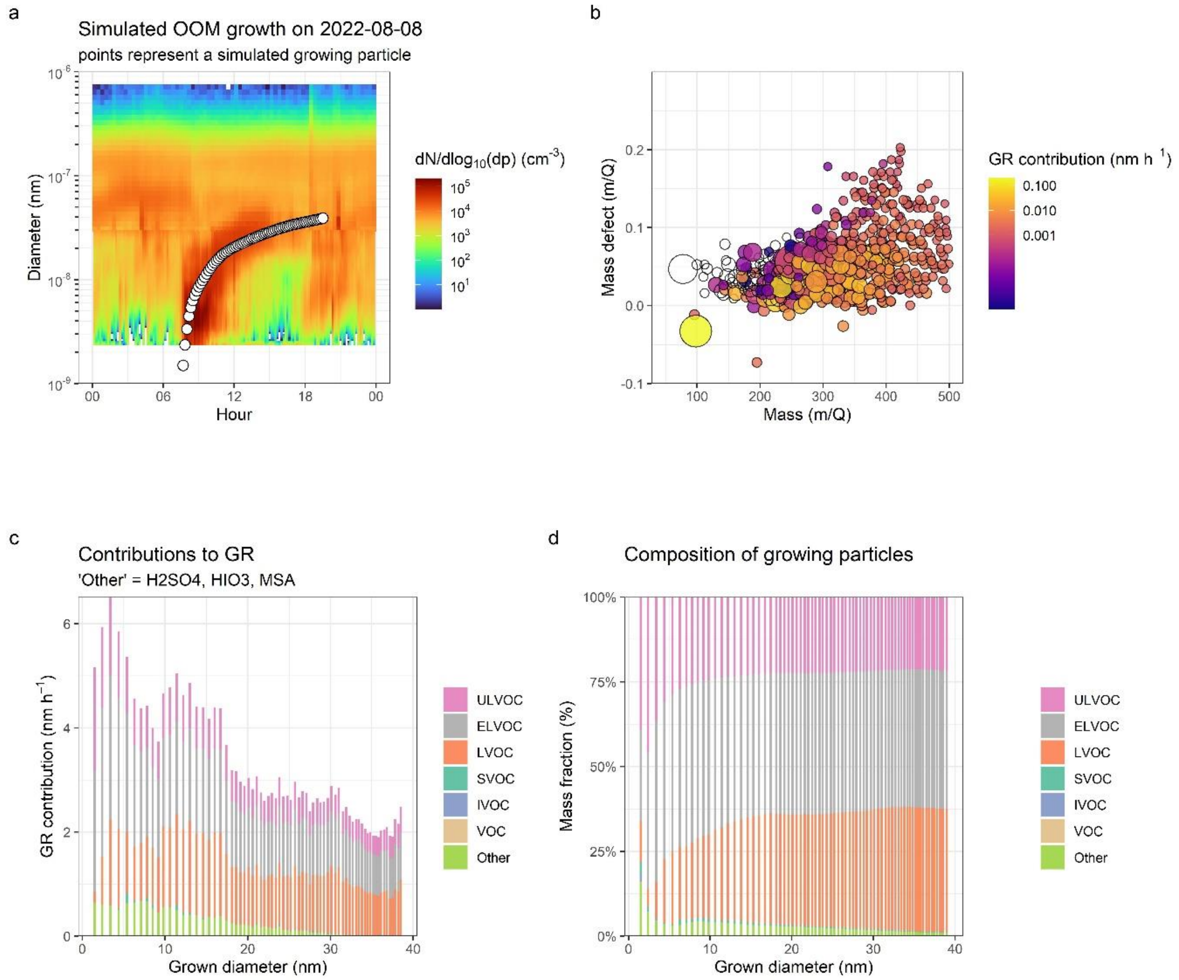

Figure S8: Example simulated particle growth on 2022-08-08, showing the time evolution of the PNSD with the simulated growth overlaid (top left), the mass defect coloured by the contribution to growth rates (top right), contribution of different species to the growth rate at each diameter (bottom left), and the resultant composition of the growing particle (bottom right).

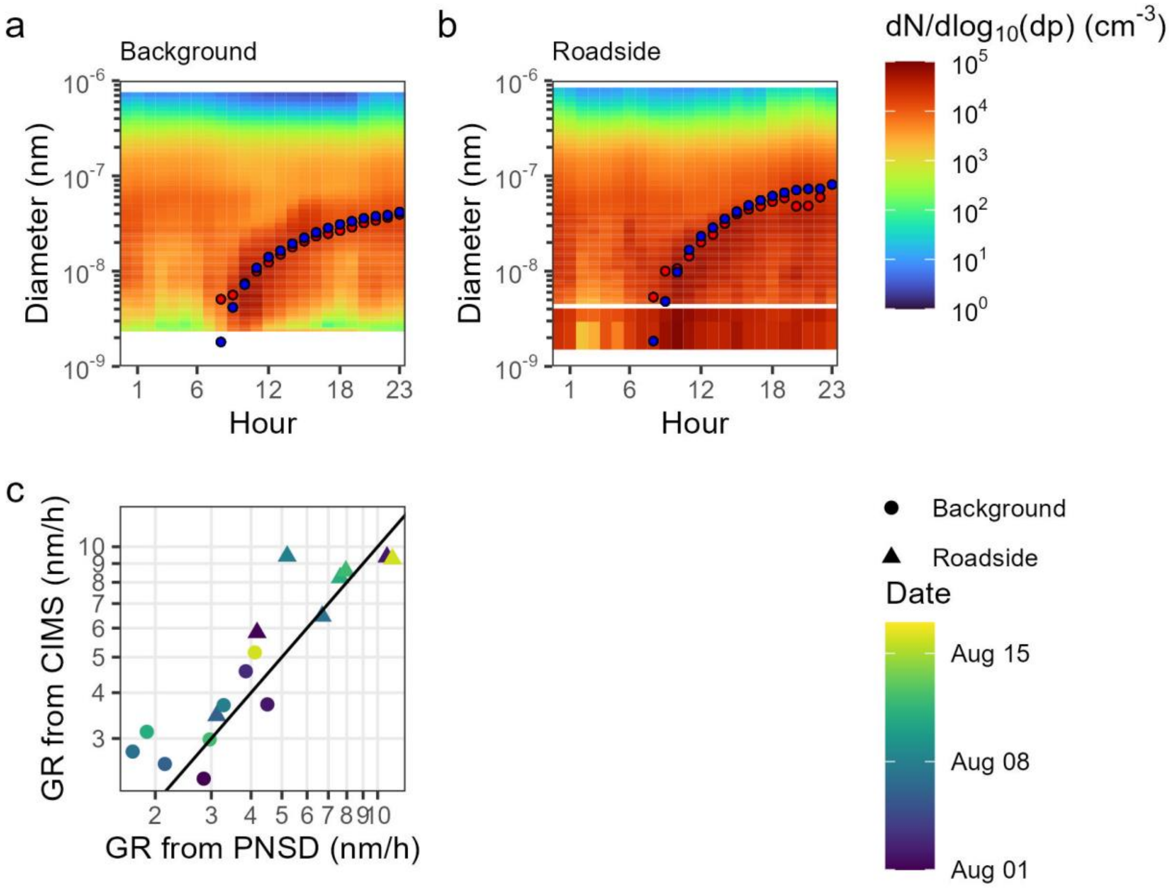

Figure S9: GRs from fitting modes to the average of MPSS data (red) and from the calculated GR from the CIMS data (blue) for the background and roadside sites (a,b). Average of all NPF events shown. Scatterplot of GRs from 7 – 25 nm from both methods in (c). Black line shows the 1:1 ratio.

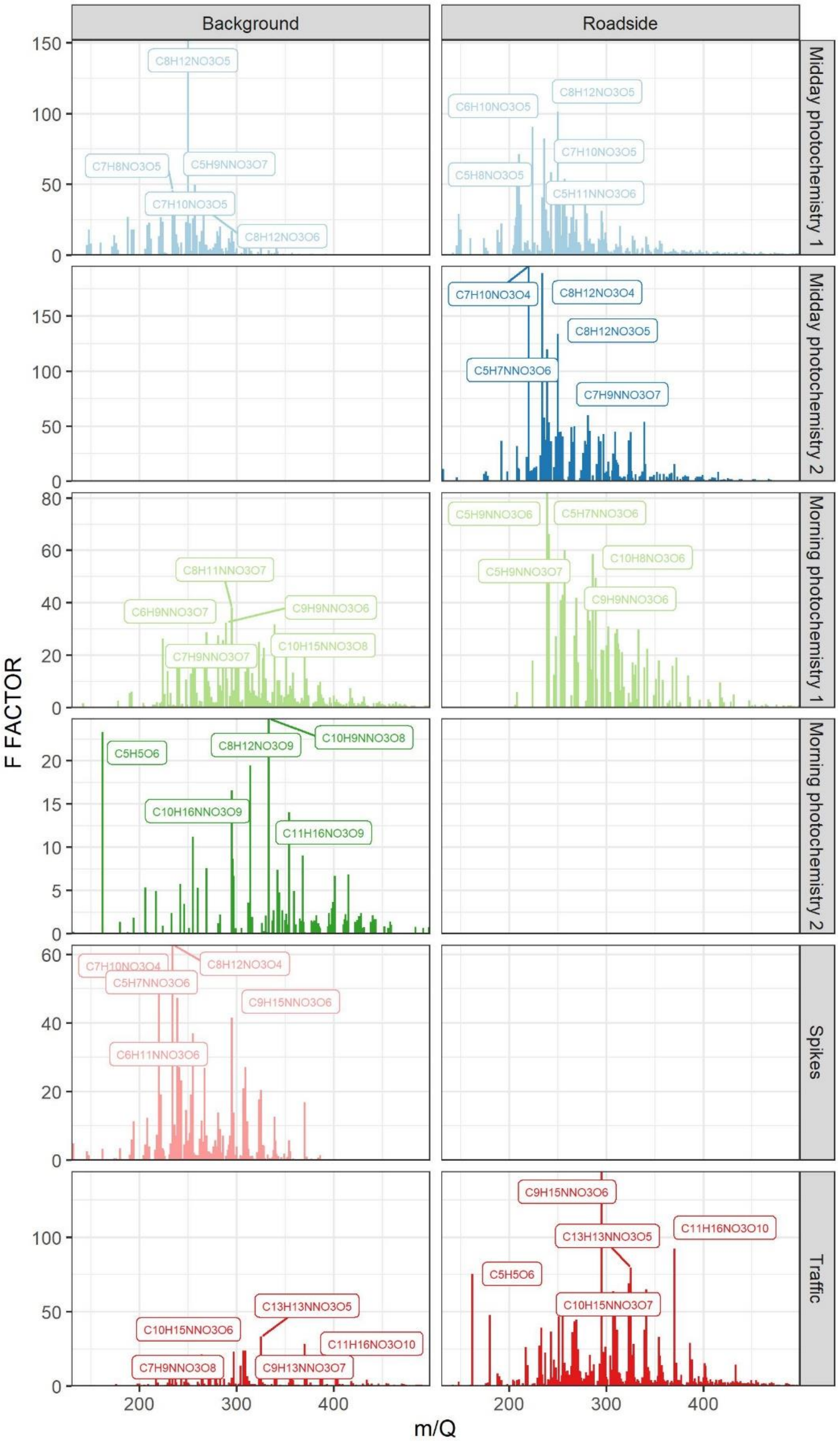

Figure S10: Average mass spectrum per factor per site

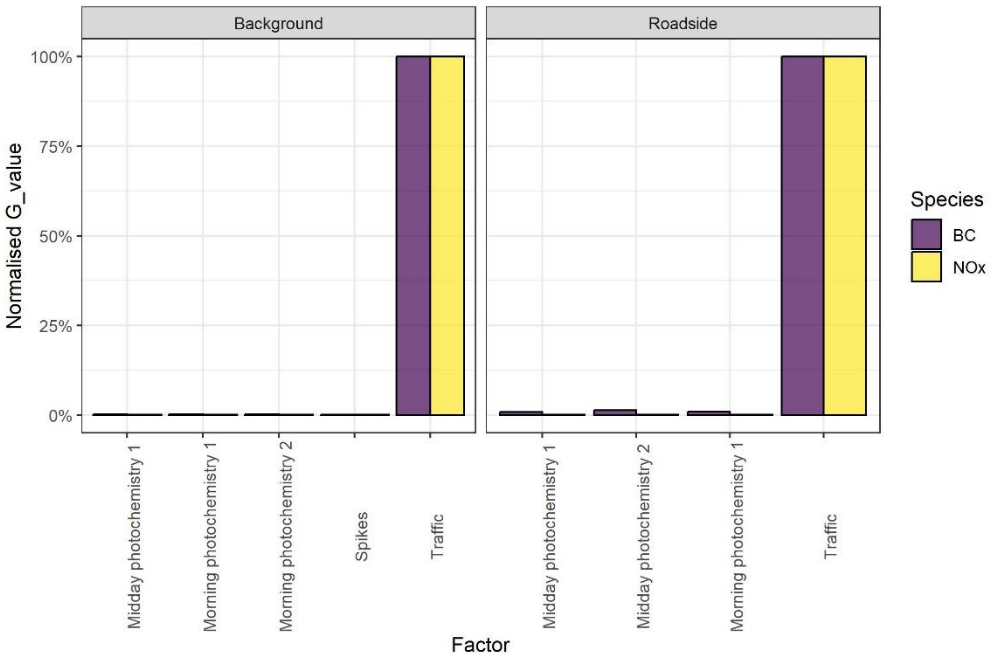

Figure S11: Association of BC and NO_x with each PMF factor at each site

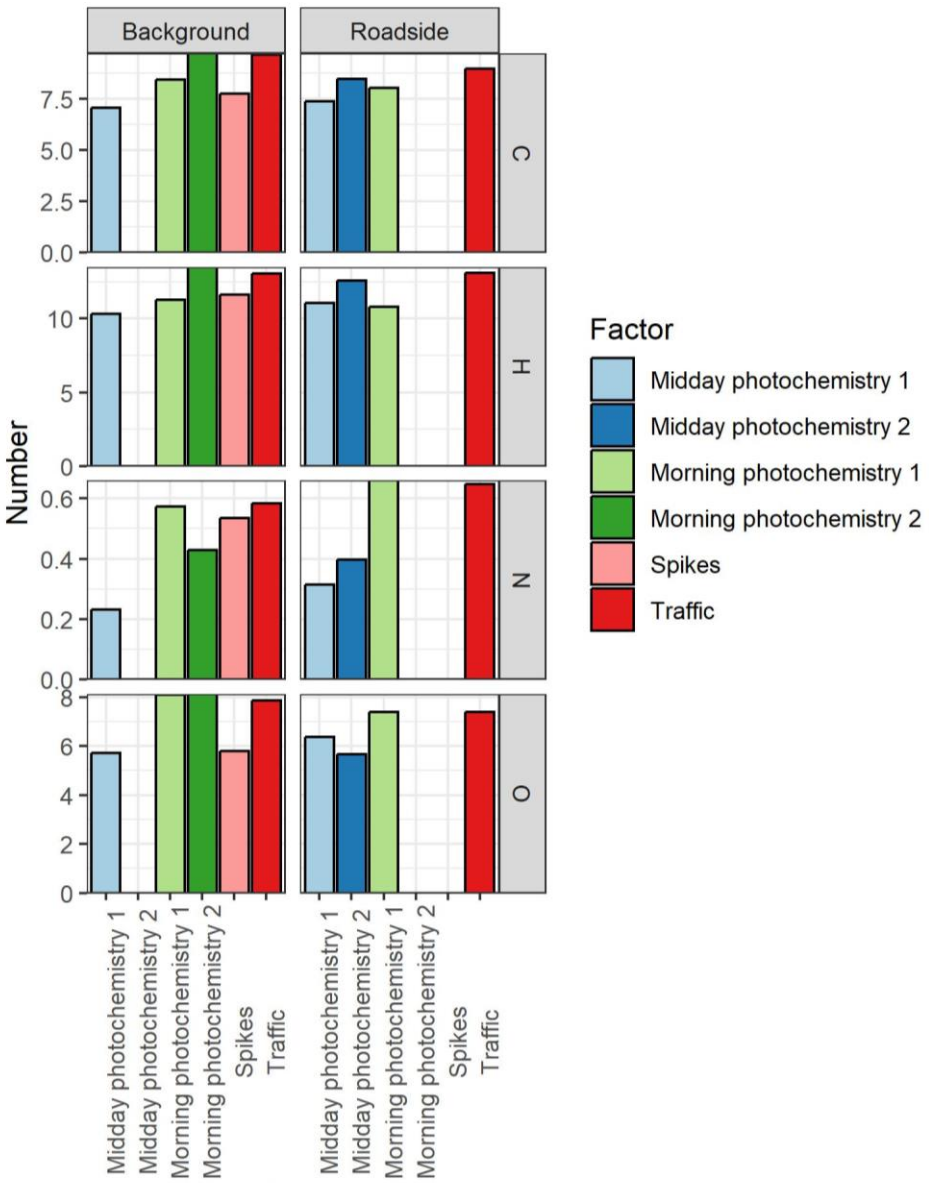

Figure S12: Average signal-weighted carbon, hydrogen, nitrogen, and oxygen numbers per PMF factor per site

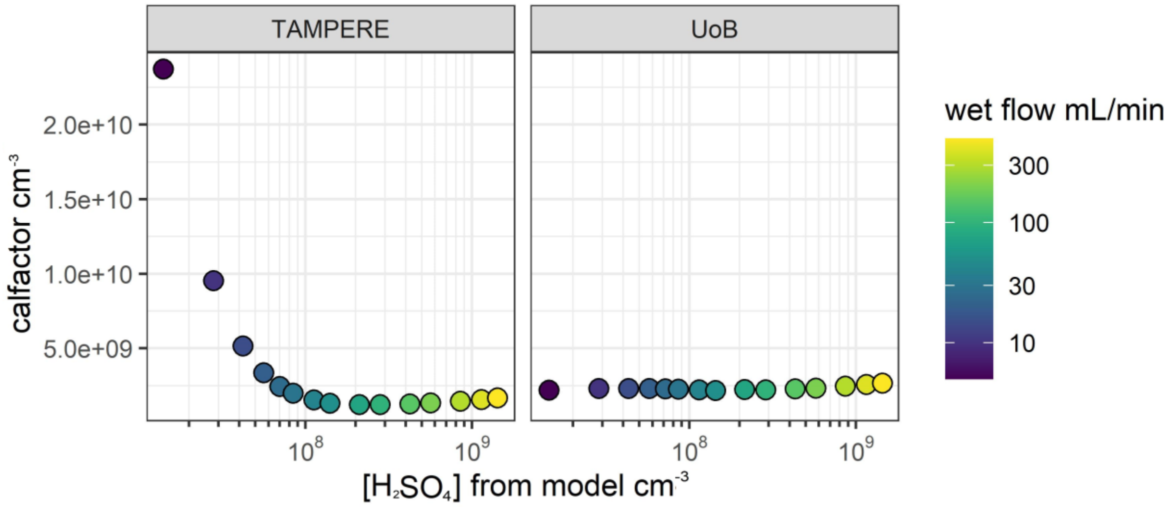

Figure S12: Calibration run results for both instruments.

(1) Kürten, A.; Rondo, L.; Ehrhart, S.; Curtius, J. Calibration of a Chemical Ionization Mass Spectrometer for the Measurement of Gaseous Sulfuric Acid. *The Journal of Physical Chemistry A* **2012**, *116* (24), 6375-6386. DOI: 10.1021/jp212123n.
(2) Kulmala, M.; Petäjä, T.; Nieminen, T.; Sipilä, M.; Manninen, H. E.; Lehtipalo, K.; Dal Maso, M.; Aalto, P. P.; Junninen, H.; Paasonen, P.; et al. Measurement of the nucleation of atmospheric aerosol particles. *Nature Protocols* **2012**, *7* (9), 1651-1667. DOI: 10.1038/nprot.2012.091.
(3) Lee, K. W.; Chen, H. Coagulation Rate of Polydisperse Particles. *Aerosol Science and Technology* **1984**, *3* (3), 327-334. DOI: 10.1080/02786828408959020.
(4) Qiao, X.; Yan, C.; Li, X.; Guo, Y.; Yin, R.; Deng, C.; Li, C.; Nie, W.; Wang, M.; Cai, R.; et al. Contribution of Atmospheric Oxygenated Organic Compounds to Particle Growth in an Urban Environment. *Environ Sci Technol* **2021**, *55* (20), 13646-13656. DOI: 10.1021/acs.est.1c02095
(5) Trostl, J.; Chuang, W. K.; Gordon, H.; Heinritzi, M.; Yan, C.; Molteni, U.; Ahlm, L.; Frege, C.; Bianchi, F.; Wagner, R.; et al. The role of low-volatility organic compounds in initial particle growth in the atmosphere. *Nature* **2016**, *533* (7604), 527-531. DOI: 10.1038/nature18271
(6) Hritz, A. D.; Raymond, T. M.; Dutcher, D. D. A method for the direct measurement of surface tension of collected atmospherically relevant aerosol particles using atomic force microscopy. *Atmospheric Chemistry and Physics* **2016**, *16* (15), 9761-9769. DOI: 10.5194/acp-16-9761-2016.
(7) Paatero, P.; Tapper, U. Positive matrix factorization: A non-negative factor model with optimal utilization of error estimates of data values. *Environmetrics* **1994**, *5* (2), 111-126, https://doi.org/10.1002/env.3170050203. DOI: https://doi.org/10.1002/env.3170050203
(8) Rivas, I.; Beddows, D. C. S.; Amato, F.; Green, D. C.; Järvi, L.; Hueglin, C.; Reche, C.; Timonen, H.; Fuller, G. W.; Niemi, J. V.; et al. Source apportionment of particle number size distribution in urban background and traffic stations in four European cities. *Environment International* **2020**, *135*, 105345. DOI: https://doi.org/10.1016/j.envint.2019.105345.
(9) Mehra, A.; Canagaratna, M.; Bannan, T. J.; Worrall, S. D.; Bacak, A.; Priestley, M.; Liu, D.; Zhao, J.; Xu, W.; Sun, Y.; et al. Using highly time-resolved online mass spectrometry to examine biogenic and anthropogenic contributions to organic aerosol in Beijing. *Faraday Discussions* **2021**, *226*, 382-408. DOI: 10.1039/d0fd00080a.
(10) Birmili, W.; Weinhold, K.; Rasch, F.; Sonntag, A.; Sun, J.; Merkel, M.; Wiedensohler, A.; Bastian, S.; Schladitz, A.; Löschau, G.; et al. Long-term observations of tropospheric particle number size distributions and equivalent black carbon mass concentrations in the German Ultrafine Aerosol Network (GUAN). *Earth System Science Data* **2016**, *8* (2), 355-382. DOI: 10.5194/essd-8-355-2016.
